# Mental health and its wider determinants in young people in the UK during 12 months of the COVID-19 pandemic: repeated cross-sectional representative survey

**DOI:** 10.1192/bjo.2024.726

**Published:** 2024-12-05

**Authors:** Olivier Y. Rouquette, Dana Dekel, Abdul-Moiz Siddiqi, Catherine Seymour, Lauren Weeks, Ann John

**Affiliations:** Swansea University Medical School, Swansea, UK; Leaders Unlocked, London, UK; Mental Health Foundation, London, UK

**Keywords:** Adolescents, anxiety, depression, loneliness, COVID-19

## Abstract

**Background:**

The COVID-19 pandemic posed an unprecedented global challenge, with past evidence suggesting negative psychological effects with the additional concern that social and physical restrictions might disproportionately affect adolescents.

**Aims:**

To explore mental health and its wider determinants in young people in the UK during 1 year of the COVID-19 pandemic (August 2020–August 2021).

**Method:**

A representative sample of 11 898 participants (48.7% female) aged between 13 and 19 years (mean = 16.1) participated in five waves of data collection. Using validated self-reported questionnaires for loneliness, anxiety and depression, this survey measured the extent and nature of the mental health impacts of the coronavirus pandemic and help-seeking behaviours, and changes over time.

**Results:**

Young people experienced higher levels of anxiety during the summer and fall 2020, followed by higher levels of depression during the winter 2020–2021, with loneliness gradually increasing then peaking during the spring and summer of 2021. Young people who were older, female, with pre-existing mental-health issues and experiencing financial difficulties were at higher risk of anxiety, depression and loneliness. Help-seeking behaviours reduced the risk of depression and loneliness.

**Conclusions:**

The COVID-19 pandemic had substantial impact on young people, whether on their mental health, their social contacts and interactions or their perspective on what the future holds for them. Young people strongly advocated for better teacher training, and a better integration of mental health services, particularly within their schools.

The COVID-19 pandemic posed an unprecedented global challenge and there is on-going debate regarding the short- and long-term impacts of associated restrictions on the mental health of children and adolescents. The public health response required a complex balance between controlling the spread of the virus and burden on healthcare with any unintended consequences of interventions, such as economic impacts and social isolation, for example, from school closure. The pandemic occurred in the context of already worsening mental health of children and young people in the UK with anxiety, depression, self-harm and suicide increasing over the previous decade^1^ – as well inadequate provision of mental health services and broader social initiatives.

During the first few weeks of the pandemic, in March 2020, global organisations and mental health charities identified the need to address the mental health consequences and mitigate them both during and after pandemic conditions.^2,3^ Some argued that mental health interventions ought to be officially integrated into emergency response plans.^4^ These calls were not baseless. Past evidence suggests negative psychological effects of quarantine, including post-traumatic stress symptoms, confusion and anger.^5^ Stressors included longer quarantine duration, infection fears, frustration, boredom, inadequate supplies, inadequate information, financial loss and stigma. Moreover, studies measuring the impact of school closures during the pandemic found that 18–60% of the children and young people scored above thresholds suggesting risk of psychological distress, particularly anxiety and depression symptoms, as a direct consequence to school closures (see, e.g. Viner et al^6^).

For this study, we focused specifically on young people. Companionship and social interactions are vital for children and young people's social and emotional development and well-being,^7,8^ hence the concern that social and physical restrictions related to COVID-19 might disproportionately affect adolescents. Despite this, studies focusing on trends in mental health in adolescents during the pandemic are scarce with even fewer including representative samples.^9–11^ One study showed average adolescent self-reported symptoms across domains (behavioural, attentional and emotional) and parent-reported emotional symptoms over time.^12^ However, the highest levels of adolescent reported symptoms were when high levels of restrictions were in place and schools were closed to most children. Another study showed that adolescents’ experiencing emotional difficulties pre-pandemic had the worst outcomes during the lockdown period.^9^ Furthermore, disproportionate effects were evident in families with low incomes throughout the pandemic.^10^ The present study adds to the understanding by using both a representative sample and validated questionnaires for loneliness, anxiety and depression.

In this study, using logistic regression, we aimed to explore mental health over time during the pandemic in adolescents and young people as well as their broader social contexts and experiences.

## Method

### Ethics

Following ethical approval by Swansea University Research Ethic Subcommittee (REC 2020–030), participants were sampled through the YouGov polling service,^13^ a UK-based international research data analytics group with a panel of over 11 million global users. This panel represents all age groups, ethnicities and socioeconomic groups, allowing for a nationally representative sample to be accessed. The YouGov survey clearly signposted to relevant helplines and sources of information if participants experienced distress when completing the questionnaires.

### Study design

This was a cross-sectional panel survey conducted over five waves of data collection during the course of 1 year in representative samples of young people in the UK population.

The survey measured the extent and nature of the mental health impacts of the coronavirus pandemic and help-seeking behaviours, as well as changes over time. The first wave (W1) of data collection occurred from 24 August 2020 to 8 September 2020, followed by a second wave (W2) from 17 November 2020 to 1 December 2020, a third wave (W3) from 25 February 2021 to 11 March 2021, a fourth wave (W4) from 24 May 2021 to 15 June 2021 and a fifth wave (W5) from 26 August 2021 to 16 September 2021.

### Study population

This study incorporated young people aged 13–19 years from across the UK, both male and female, who were able to understand, read and speak English as well as have the capacity to give consent to take part in the study. For participants aged 16 years and over written consent was sought and obtained before study participation. For participants below the age of 16 years, parental written consent was sought and obtained through YouGov prior to participation.

### Participant recruitment and data collection procedures

At each wave of data collection, the online questionnaires were co-designed and piloted by the research team with a focus group of young people recruited through Leaders Unlocked (http://leaders-unlocked.org/). Participants suggested topics and subsequently offered feedback on wording, clarifications and amendments to questions. Their feedback was reviewed by the research team and, where possible (e.g. validated questionnaires retained fidelity), suggestions were included in the survey. As such, young people from Leaders Unlocked were involved in co-designing the policy questions at W3–5. One young person from Leaders Unlocked is a co-author (A.-M.S.).

The final survey version was administered to members of the YouGov Plc UK panel of over a million individuals who have agreed to take part in surveys.^13^ Emails were sent to panellists selected at random from the base sample. The email invited them to take part in a survey and provides a generic survey link. Once a panel member clicked on the link, they were sent to the survey, based on the sample definition and quotas (non-probability sampling). Invitations to surveys did not expire and respondents were sent to any available survey. Sample quotas were based on age, gender, education level, social grade and the UK's four nation population profile. This profile was obtained from Office for National Statistics (ONS) census data and the National Readership Survey.^14^ Respondents were different in each wave but were sampled from the same panel and representative of the UK population aged between 13 and 19 years.

### Measures

#### Outcome variables

##### Loneliness

Loneliness was assessed using the UCLA (University of California, Los Angeles) three-item loneliness scale.^15^ Participants were asked how often they felt that they had no one to talk to, how often they felt left out and how often they felt alone during the past three month. Each item was scored 1–3 (*1 for hardly ever, 2 for some of the time, 3 for often*). Using a cut-off point of 6+, scores were grouped into ‘not lonely’ (people with a score of 3–5) and ‘lonely’ (people with a score of 6–9).^16,17^ The psychometric properties of the scale (i.e. reliability), such as validity with similar populations, are well documented.^15,16,18^ The internal consistency (Cronbach's alpha: α = 0.86) for the present study was satisfactory.

##### Anxiety

Anxiety was assessed using the generalised anxiety disorder seven-item scale (GAD-7), adapted for use in adolescents.^19^ Participants were asked their frequency of experiencing each item (e.g. *feeling nervous, anxious, or on edge*; *worrying too much about different things*) during the past 2 weeks. Each item was scored 0–3 (from *0 for not all* to *3 for nearly every day*). A cut-off point of 10 + was used to define clinically relevant anxiety.^20–22^ The psychometric properties of the GAD-7 have been documented in the general population,^19^ with more recent studies demonstrating similar properties among young people.^23–25^ In the present study, the internal consistency (Cronbach's alpha: α = 0.93) was also satisfactory.

#### Depression

Depression severity was assessed using the patient health questionnaire eight-item scale (PHQ-8^26^). Participants were asked their frequency of experiencing each item (e.g. *feeling down, depressed, irritable or hopeless*; *feeling tired or having little energy*) during the past 2 weeks. Items were scored between 0 and 3 for each item (from *0 for not all* to *3 for nearly every day*). A cut-off point of 10 + was used to define clinically relevant depression.^26,27^ The psychometric properties of the PHQ-8 are well documented in the general population,^26^ with further work demonstrating that the PHQ-8 was appropriate to screen for depression among adolescents and young people.^28^ The reliability in the current study was also satisfactory (Cronbach's alpha: α = 0.92).

#### Covariates

##### Sociodemographics

Demographic variables included the categorical variables of gender (male or female), age (13–17 and 18–19), region (North/Scotland, Midlands/Wales, East England, London, and South England) and ethnicity. Participants were asked if they had been diagnosed with a mental health or emotional disability (e.g. mood disorder, schizophrenia, etc.) that had a substantial and long-term impact on their day-to-day life (yes/no). Participants were also asked to respond to various questions pertaining to the impact the COVID-19 pandemic had on their life, such as health and economic consequences for them and their families as a result of the pandemic, across five waves of data collection.

##### Help-seeking behaviours

Participants were asked which people or service they would feel confident getting help from if they needed help with their emotional or mental health. Participant were given multiple-choice selection of the following options: *family and/or friends*, *a website*, *social media*, *a helpline*, *a web chat or text service*, *teachers or other school staff*, *their doctor/GP* [general practitioner], *a mental health team in their area*, *school counselling*, *none of these*, *don't know* or *prefer not to say*.

##### Policy questions

At W3 (25 February 2021), W4 (24 May 2021) and W5 (26 August 2021) we asked participants their opinion on what could be done to improve their mental health as coronavirus restrictions ease. Participants responded with a multiple-choice selection of various propositions at W3 and W4, and with a single choice at W5 (Supplementary Tables 3–5 available at https://doi.org/10.1192/bjo.2024.726).

### Data analysis

All analyses were performed with R-statistics (version 3.6.1.) through R-Studio (RStudio Team, Boston, Massachusetts, USA; http://www.rstudio.com/). For each wave of data collection, sample weighting was incorporated into statistical analysis to obtain representative UK estimates. Descriptive statistics (frequencies, means and 95% confidence intervals) were presented for outcome measures and explanatory factors for each of the five survey waves.

We used weighted crosstabulation tables with adjusted Wald corrections^29^ allowing for clustering and stratification in the data to evaluate changes in loneliness, anxiety and depression across the five waves of data collection. Logistic regression were carried out with robust standard error,^30^ and with revised weight following recommendations from Korn and Graubard^31^ for multiple surveys. Logistic regressions were carried out separately for anxiety, depression and loneliness accounting for time (W1–W5 of data collection), ethnicity (White versus ethnic minority), region (North/Scotland, Midlands/Wales, East England, London and South England), age (13–17 versus 18–19 years old), gender (male versus female), previous history of mental health condition (0/1), financial difficulties (categorical) and social media uses (from less than 1 h up to more than 6 h, help-seeking behaviour). We subsequently used stepwise regression as an exploratory data analysis to select the most useful predicting variables for each model.^32^ The stepwise procedure was conducted backward and forward, with time (W1–W5) always included in the models, and with Akaike information criteria (AIC) to evaluate the fit of the model. The level of statistical significance was set at *P* = 0.05. We also checked underlying assumptions such as multicollinearity (variance inflation factor (VIF)) and influential values (Cook's distance) for each model.

## Results

### Participant characteristics

In total, 11 898 participants (48.7% female, 51.3% male) aged between 13 and 19 years (mean = 16.1, s.d. = 0.2) participated in the five waves of data collection (W1: *n* = 2375, W2: *n* = 2395, W3: *n* = 2368, W4: *n* = 2349, W5: *n* = 2411). Participants were from the North/Scotland (32.3%), the South (22.7%), the Midlands/Wales (21.9%), London (13.5%) and the East (9.6%). In the present sample, 88.3% of participants were White, and 11.7% from ethnic minority groups. In total, 9.2% (95% CI = 8.7–10.0%) of participants reported pre-existing mental health issues.

### Coronavirus infections

Coronavirus infections rates for participants ranged from 0.7% (95% CI = 0.4–1.1%) of positive tests at W1 (24 August 2020) up to 12.1% (95% CI = 10.8–13.6%) of positive tests at W5 (26 August 2021). Having someone in the household testing positive ranged from 2.7% (95% CI = 2.0–3.4%) at W1 (24 August 2020) to 16.2% (95% CI = 14.6–17.8%) at W5 (26 August 2021) (see Supplementary Table 1 for full results).

### Health consequences of coronavirus infection

**Fig. 1:**
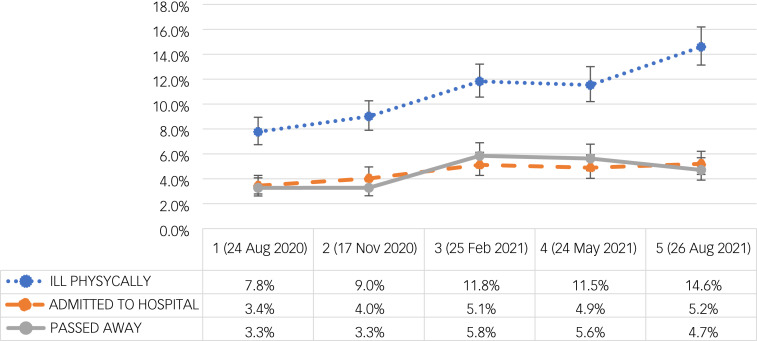
Health consequences of coronavirus: percentage of participants reporting being ill physically owing to coronavirus (blue), that someone in their close family was admitted to hospital (orange) or died (grey) owing to coronavirus infection with 95% CI (vertical lines) throughout five waves of data collection from 1 (24 August 2020) to 5 (26 August 2021).

The proportion of participants reporting that they had been physically ill owing to coronavirus increased from 7.7% (95% CI = 6.7–8.9%) at W1 (24 August 2020) to 14.6% (95% CI = 13.1–16.1%) at W5 (26 August 2021): *F*(4; 11 894) = 15.7, *P* < 0.01. The proportion of participants reporting that someone in their family had been admitted to hospital owing to coronavirus also significantly varied with time with a proportion of 3.5% (95% CI = 2.8–4.2%) at W1 (24 August 2020) to 5.2% (95% CI = 4.3–6.2%) at W5 (26 August 2021): *F*(4; 11 894) = 3.3, *P* = 0.009. The proportion of participants reporting that someone in their family had passed away owing to coronavirus also varied with time, with proportion ranging from 3.3% (95% CI = 2.6–4.0%) at W1 (24 August 2020) to 5.6% (95% CI = 4.6–6.7%) at W4 (24 May 2021): *F*(4, 11 894) = 8.0, *P* < 0.001 (Fig. 1).

### Economic consequences of coronavirus

**Fig. 2:**
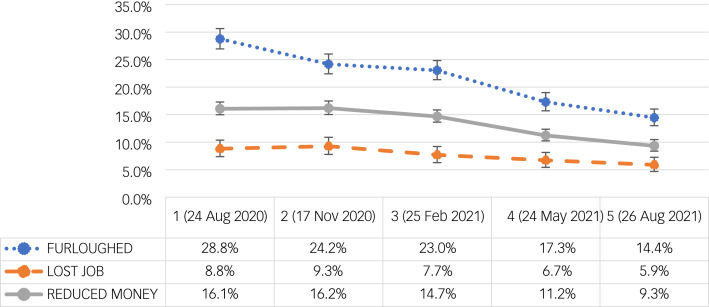
Economic consequences of coronavirus: percentage of participants reporting that someone in their close family had been furloughed (blue), lost their job (orange) or that they had reduced money (grey) owing to coronavirus infection with 95% CI (vertical lines) throughout five waves of data collection from 1 (24 August 2020) to 5 (26 August 2021).

Many employers were unable to operate (either partially or fully) during the pandemic, so the UK Government set up the Coronavirus Job Retention Scheme (CJRS), referred to as ‘furlough’. The scheme provided grants to employers so they could retain and continue to pay staff during coronavirus related lockdowns, by furloughing employees at up to 80% of their wages. The proportion of individuals reporting that someone in their close family had been ‘furloughed’ decreased significantly from 28.8% (95% CI = 26.9–30.6%) at W1 (24 August 2020) to 14.4% (95% CI = 13.0–16.0%) at W5 (26 August 2021): *F*(4.0; 47 501.3) = 41.4, *P* < 0.001. Participants reported that someone in their close family had lost their job peaked at W2 (17 November 2020) with 9.3% (95% CI = 8.1–10.6%), down to 5.9% (95% CI = 4.9–7.0%) at W5 (26 August 2021): *F*(3.9; 47 422.7) = 6.1, *P* < 0.001 (Fig. 2).

### Loneliness

Participants scores of loneliness were consistently higher than 50% across the five waves of data collection (Table 1). The results of the logistic regression showed that the rate of loneliness varied with time, with participants from W4 (24 May 2021) and W5 (26 August 2021) of data collection more likely to report loneliness (odds ratio = 1.2 and odds ratio = 1.2, respectively) compared with participants from W1 of data collection (24 August 2020). Participants aged 18 and over (odds ratio = 1.6), of female gender (odds ratio = 1.3), with pre-existing mental health issues (odds ratio = 1.7), reporting either a lot of financial difficulties (odds ratio = 2.1), a little (odds ratio = 1.5) or not knowing if they had financial difficulties (odds ratio = 1.4) were also more likely to experience loneliness. Participants reporting using social media for 1–4 h (odds ratio = 1.4), 4–6 h a day (odds ratio = 1.8) and for more than 6 h a day (odds ratio = 1.4) were also more likely to experience loneliness compared with participants reporting no social media use at all. Participants reporting feeling confident in getting help for their emotional well-being were less likely to report loneliness (odds ratio = 0.7) compared with participants not being confident in seeking help (Table 2). The goodness of fit of the model was AIC = 153.3. Exploratory stepwise analysis led to an improved fit of the model of AIC = 142.0 by removing the ethnicity and region variables (Supplementary Table 2). The model's assumptions were met with low correlations between predictor variables (VIF < 4) and no influential outliers.

**Table 1:** Proportion of participants (95% CI) above the cut-off scores for anxiety (generalised anxiety disorder seven-item scale (GAD-7)), depression (patient health questionnaire eight-item scale (PHQ-8)) and loneliness (University of California, Los Angeles (UCLA)) throughout five waves of data collection from wave 1 (24 August 2020) to wave 5 (26 August 2021)

Variable	1	2	3	4	5
	24 Aug 2020 (%)	17 Nov 2020 (%)	25 Feb 2021 (%)	24 May 2021 (%)	26 Aug 2021 (%)
Anxiety disorder (GAD-7 ≥ 10)	23.1 (21.5–25.0)	25.7 (23.9–28.0)	23.5 (21.8–25.0)	21.4 (19.7–23.0)	20.4 (18.7–22.0)
Depressive disorder (PHQ-8 ≥ 10)	25.8 (24.1–28.0)	30.1 (28.1–32.0)	31.4 (29.4–33.0)	26.0 (24.1–28.0)	24.3 (22.5–26.0)
Loneliness (UCLA ≥ 6)	50.8 (48.8–53.0)	52.7 (50.5–55.0)	53.6 (51.5–56)	53.9 (51.7–56)	52.7 (50.6–55.0)

**Table 2:** Results of the weighted logistic binomial regression with robust standard errors (heteroskedasticity-consistent estimator (HC3)) for anxiety, depression and loneliness with odds ratios, 95% CI and *P*-value, controlling for ethnicity (White versus ethnic minority group) and regions in the UK

	Anxiety (GAD-7 ≥ 10)			Depression (PHQ-8 ≥ 10)			Loneliness (UCLA ≥ 6)		
Variables	Odds ratios	95% CI	*P*	Odds ratios	95% CI	*P*	Odds ratios	95% CI	*P*
Wave 1 – 24 Aug 2020 (Ref.)									
Wave 2 – 17 Nov 2020	1.0	0.8–1.3	0.687	**1**.**2**	**1.0–1.5**	**0**.**037**	1.0	0.9–1.2	0.639
Wave 3 – 25 Feb 2021	**0**.**8**	**0.7–1.0**	**0**.**031**	**1**.**6**	**1.3–1.9**	**<0**.**001**	1.1	0.9–1.2	0.245
Wave 4 – 24 May 2021	0.8	0.7–1.0	0.070	1.1	0.9–1.3	0.389	**1**.**2**	**1.0–1.4**	**0**.**009**
Wave 5 – 26 Aug 2021	0.9	0.7–1.1	0.190	0.9	0.8–1.2	0.789	**1**.**2**	**1.0–1.4**	**0**.**005**
13–17 years old (Ref.)									
18–19 years old	1.3	1.1–1.5	**<0**.**001**	2.0	1.8–2.3	**<0**.**001**	1.6	1.4–1.8	**<0**.**001**
Male (Ref.)									
Female	1.4	1.2–1.6	**<0**.**001**	1.3	1.1–1.5	**<0**.**001**	1.3	1.2–1.4	**<0**.**001**
No mental health issue (Ref.)									
Pre-existing mental health issues	3.2	2.6–3.9	**<0**.**001**	2.7	2.2–3.4	**<0**.**001**	1.7	1.4–2.1	**<0**.**001**
No financial difficulties (Ref.)									
Financial difficulties: a lot	1.8	1.4–2.2	**<0**.**001**	2.1	1.7–2.6	**<0**.**001**	2.1	1.7–2.5	**<0**.**001**
Financial difficulties: a little	1.1	0.9–1.3	0.141	1.5	1.3–1.7	**<0**.**001**	1.5	1.4–1.7	**<0**.**001**
Financial difficulties: don't know	0.9	0.8–1.1	0.670	1.3	1.1–1.5	**0**.**002**	1.4	1.2–1.6	**<0**.**001**
Financial difficulties: prefer not to say	1.5	1.0–2.3	**0**.**034**	0.9	0.6–1.4	0.807	1.3	1.0–1.8	0.076
No social media use (Ref.)									
Social media: <1 h	0.7	0.5–0.9	**0**.**004**	1.0	0.8–1.4	0.730	1.1	0.9–1.3	0.221
Social media: 1–4 h	0.7	0.6–0.9	**0**.**012**	1.1	0.8–1.4	0.396	1.4	1.2–1.7	**<0**.**001**
Social media: 4–6 h	0.8	0.6–1.0	0.103	1.8	1.3–2.3	**<0**.**001**	1.8	1.46–2.15	**<0**.**001**
Social media: >6 h	0.9	0.7–1.2	0.499	2.6	2.0–3.5	**<0**.**001**	1.4	1.2–1.8	**0**.**001**
Social media: don't know	1.3	0.8–1.9	0.221	0.8	0.5–1.2	0.311	1.3	0.9–1.7	0.119
No help-seeking behaviour (Ref.)									
Help-seeking behaviour	0.9	0.8–1.1	0.607	0.7	0.6–0.7	**<0**.**001**	0.7	0.6–0.8	**<0**.**001**
Depressive disorder (PHQ-8 ≥ 10)	15.4	13.5–17.7	**<0**.**001**				4.2	3.6–4.8	**<0**.**001**
Anxiety disorder (GAD-7 ≥ 10)				15.5	13.5–17.7	**<0**.**001**	2.3	2.0–2.7	**<0**.**001**
Loneliness (UCLA ≥ 6)	2.3	2.0–2.7	**<0.001**	4.2	3.7–4.9	**<0**.**001**			
Observations	11 192			11 192			11 192		
*R*^2^ Tjur	0.438			0.480			0.218		

GAD-7, generalised anxiety disorder seven-item scale; PHQ-8, patient health questionnaire eight-item scale; UCLA, University of California, Los Angeles.

Figures shown in bold: *P* < 0.05.

### Anxiety

The proportion of participants with anxiety symptoms peaked at W2 with 25.7% (95% CI = 23.9–27.6%) of participants having score of GAD-7 ≥ 10. The rate of participants with anxiety symptoms subsequently decreased with time 20.4% (95% CI = 18.7–22.1%) at W5. Overall, changes in participants’ anxiety were significant across the five waves of data collection: *F*(4; 11 894) = 5.0, *P* < 0.001 (Table 1).

The results of the logistic regression showed that the rate of anxiety symptoms varied with time, with participants from W3 of data collection (25 February 2021) less likely to report anxiety symptoms (odds ratio = 0.8) compared with participants from W1 (24 August 2020). Participants aged 18 and over (odds ratio = 1.3), of female gender (odds ratio = 1.4), with pre-existing mental health issues (OR = 3.2), reporting either high levels of financial difficulties (odds ratio = 1.8) or preferring not to report financial difficulties (odds ratio = 1.5) were more likely to experience anxiety symptoms. Participants reporting using social media for less than 1 h a day (odds ratio = 0.7) or for 1–4 h a day (odds ratio = 0.7) were also less likely to experience anxiety symptoms compared with participants reporting no social media use at all (Table 2). The goodness of fit of the model was AIC = 153.3. Exploratory stepwise analysis led to an improved fit of the model of AIC = 142.0 by removing the ethnicity and region variables (Supplementary Table 2). The model's assumptions were met with low correlations between predictor variables (VIF < 4) and no influential outliers.

### Depression

The proportion of participants with depressive symptoms peaked at W3 (25 February 2021) of data collection with 31.4% (95% CI = 29.4–33.3%) of participants having scores of PHQ-8 ≥ 10. This rate of depressive symptoms then gradually decreased to 24.3% (95% CI = 22.5–26.2%) at W5 of data collection. Overall, changes in participants’ depressive symptoms were significant across the five waves of data collection: *F*(4; 11 894) = 9.5, *P* < 0.001 (Table 1).

The results of the logistic regression showed that the rate of depressive symptoms varied with time, with participants from W2 (17 November 2020) and W3 (25 February 2021) of data collection more likely to report depressive symptoms (odds ratio = 1.2 and odds ratio = 1.6, respectively) compared with participants from W1 of data collection (24 August 2020). Participants aged 18 and over (odds ratio = 2.0), of female gender (odds ratio = 1.3), with pre-existing mental health issues (odds ratio = 2.7), reporting either a lot of financial difficulties (odds ratio = 2.1), a little (odds ratio = 1.5) or not knowing if they had financial difficulties (odds ratio = 1.3) were also more likely to experience depressive symptoms. Participants reporting using social media for 4–6 h a day (odds ratio = 1.8) and for more than 6 h a day (odds ratio = 2.6) were also more likely to experience depressive symptoms compared with participants reporting no social media usage at all. Participants reporting feeling confident in getting help for their emotional well-being were also less likely to report depressive symptoms (odds ratio = 0.7) compared with participants not being confident in seeking help (Table 2). The goodness of fit of the model was AIC = 82.0. Exploratory stepwise analysis led to an improved fit of the model of AIC = 72.4 by removing the ethnicity and region variables (Supplementary Table 2). The model's assumptions were met, with low correlations between predictor variables (VIF < 4) and no influential outliers.

### Help-seeking behaviours

**Fig. 3:**
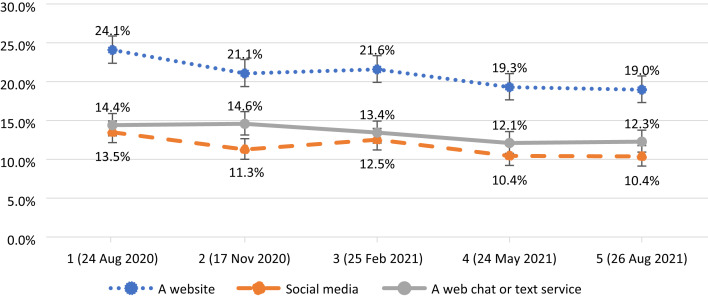
Percentage of participants feeling confident in getting help from a website (blue), social media (orange) and a web chat or service (grey) with 95% CI (vertical lines) throughout five waves of data collection from 1 (24 August 2020) to 5 (26 August 2021).

Approximately 85% of participants reported feeling confident getting help from a least one person or service, with this proportion not significantly changing throughout the five waves of data collection: *F*(4; 11 894) = 0.9, *P* = 0.455. (Table 3). However, the proportion of participants feeling confident in getting help from online services (i.e. website, social media or a web chat or text service) diminished with time: *F*(4; 11 984) = 5.4, *P* < 0.001 for website, *F*(4; 11 984) = 3.8, *P* = 0.004 for social media and *F*(4; 11 894) = 2.5, *P* = 0.04 for web chat or text service (Fig. 3).

**Table 3:** Percentage of participants reporting feeling confident getting help from people and services throughout five waves of data collection from 1 (24 August 2020) to 5 (26 August 2021)

1	2	3	4	5	Which, if any, of the following people/services would you feel confident getting help from?
24 Aug 2020 (%)	17 Nov 2020 (%)	25 Feb 2021 (%)	24 May 2021 (%)	26 Aug 2021 (%)	
68.9	67.8	68.6	67.4	69.1	Family and/or friends
24.1	21.1	21.6	19.3	19.0	A website
13.5	11.3	12.5	10.4	10.4	Social media
15.6	15.2	16.6	15.7	14.8	A helpline
14.4	14.6	13.4	12.1	12.3	A web chat or text service
28.3	28.4	27.3	28.4	28.0	Teachers or other school staff
35.8	32.2	34.0	34.5	33.9	Your doctor/GP
19.7	17.6	18.2	17.6	17.4	A mental health team in your area
21.7	22.0	21.1	21.7	21.3	School counselling
**85.1**	**84**.**9**	**83**.**7**	**83**.**4**	**84**.**1**	**At least one of the above**
6.3	6.7	6.5	5.9	5.9	None of these
7.0	6.9	6.0	6.6	6.4	Don't know
1.5	1.4	2.2	2.6	2.5	Prefer not to say

GP, general practitioner.

Figures shown in bold: *P* < 0.05.

### Policy questions

Participants provided their opinion on what could be done to benefit and improve their mental health as restrictions eased at W3 (25 February 2021), W4 (24 May 2021) and W5 (26 August 2021) of data collection. At W3 (25 February 2021), the highest ranked proposition was helping teachers to better understand and address teenagers’ mental health, followed by making it compulsory for every school to have a mental health and well-being policy (Supplementary Table 3). At W4 (24 May 2021) of data collection, participants ranked in first place the proposal to have a counsellor in every school and increasing counselling services available to young people. Participants also championed programmes to get young people into work for the first time (Supplementary Table 4). Finally, at W5 (26 August 2021) of data collection, participants ranked first again the proposition of a making it compulsory for every school to have a mental health and well-being policy. They subsequently championed the necessity to catch-up with friends and teachers rather than focusing too much on missed learning (Supplementary Table 5).

## Discussion

The COVID-19 pandemic generated multiple health, economic and social disruptions in young people's everyday lives. Our results show that levels of loneliness gradually increased with time, peaking during the spring and summer of 2021 (W4 and W5 of data collection), in parallel with the health consequences gradually increasing over time, with 14.5% of the respondents being physically ill in the summer of 2021. Young people experienced higher levels of anxiety during the summer and fall of 2020 (W1 and W2 of data collection). Interpreting this is tricky – it may be related to uncertainties regarding financial adversity, exams or university places, which were highly uncertain at the time. The negative impact on social life and activities peaked during the winter of 2020–2021 during further social restrictions and confinement, which aligns with the higher levels of depression during the winter of 2020–2021 (W2 and W3 of data collection). In addition to the temporal trends in young people's mental health, our results show several commonalities in risk factors associated with loneliness, anxiety and depression. Shared risks factors included being female (versus male), being aged 18–19 years (versus aged 13–17 years), experiencing financial difficulties, having pre-existing mental health issues and reporting higher levels of anxiety, depression or loneliness concurrently.

Higher levels of mental health issues for young people aged 18–19 years, compared with those aged 13–17 years, likely partly reflects existing trends in onset of mental health issues.^33,34^ However, the higher proportion of mental health issues reported by those aged 18–19 years (compared with younger adolescents) could also be related to uncertainties regarding their future and their transition to education, or to work.^35^ We are unable to see if this difference widened during the pandemic using our data, that is, we do not have pre-pandemic data. Not surprisingly, our models also show that the odds of loneliness, anxiety and depression were higher for individuals experiencing financial difficulties. This corresponds with other studies reporting that financial strain during COVID-19 had a bigger impact and increased risk to young people's mental health.^36,37^ Female gender was also significantly associated with higher risk of loneliness, anxiety and depression throughout the analyses; however, this phenomenon is not specific to the COVID-19 pandemic, nor an unexpected finding since higher scores for loneliness, anxiety and depression are commonly reported in the literature.^38,39^

Different risk factors were also distinctively associated with loneliness and depression, and with anxiety. For example, daily use of social media for 4 h or more was associated with an increased risk of loneliness and depression but not with an increased risk of anxiety. On the other hand, daily use of social media for less than 1 h and for 1–4 h was associated with a lower risk of anxiety than those reporting no social media use. These findings must be interpreted with caution as, in the current study, we only measured the amount of daily social media use, but not the type of usage, the reason for viewing or content viewed. Recent evidence suggests that different types of social media usage trigger positive or negative impacts, depending on the nature and circumstances of it use.^40^ For instance, Cauberghe et al^41^ presented evidence of adolescents using different social media strategies (e.g. active, social relation, humour) during the coronavirus lockdown to cope with anxiety and loneliness.

Help-seeking behaviours were related to a reduced risk of loneliness and depression, but the relationship between help-seeking behaviours and anxiety was not significant. One possible explanation is that anxiety levels rose among young people, particularly at the beginning of the COVID-19 pandemic, and that such high levels of anxiety were mainly circumstantial, with lower influence of mitigating factors such as help-seeking behaviours. Nonetheless, our results indicate that young people who felt confident in seeking help had lower levels of loneliness and depression. It is important to note that confidence in getting help in person (such as from family and friends, GPs, teachers, school counsellors or mental health teams) remained consistent across the five waves of data collection. However, young people's confidence in getting help online from a website, social media or web chat gradually decreased with time across the five waves of data collection.

While the rapid spread and the global impact of the COVID-19 pandemic was unprecedented, previous epidemics and pandemics have occurred. Research on past major pandemics (e.g. plague, cholera, influenza, severe acute respiratory syndrome (SARS), etc.) shows that the prevention and public health responses to contain such outbreaks will probably remain similar with diagnosis, identification, isolation and quarantine, protection, vaccines and drugs.^42,43^ Despite their limitations and intrinsic differences, previous research has demonstrated a positive association between mental health problems (e.g. anxiety, depression) and infectious disease epidemics compared with non-epidemic periods.^44^ More specifically, a recent comparative systematic review among Middle East respiratory syndrome (MERS), SARS and COVID-19 showed higher incidence of anxiety and depression during the COVID-19 pandemic, particularly for young people.^45^ Therefore, based on the results of the present study and in line with findings from previous studies, we can anticipate a rise in mental health difficulties among young people during a future pandemic and/or a lockdown period.

### Implications for policy and practice

We asked participants their opinion on what could be done to improve their mental health as restrictions eased to inform future policy and practice. Young people were aware of their mental health issues, were talking about them and wanted improved help and support, particularly within their schools and communities. This message aligns with the need for more integrated services at all levels, from community to primary, secondary and tertiary care settings.^46^ Participants strongly endorsed the suggestion that teachers should having a better understanding of mental health and required support and training echoing the call from the Royal Society of Medicine to better fund, support and equip teachers to promote mental health and respond to issues, including by signposting.^47^ Young people also advocated for each school to have a counsellor available, as well as mental health and well-being policies in place, which again aligns with the rationale that schools are an ideal location for young people to directly and independently access help.^47^ The majority of young people in our study said they would seek help from friends or family, so raising awareness and mental health literacy and creating easily accessible information and signposting resources at a population level should be a priority in future pandemic preparedness. Finally, and given that young people are commonly employed in sectors most affected by restrictions, such as hospitality, support for transition to employment was also regarded as important. This may be increasingly important as economic protections disappear.

### Strengths and limitations

This was a representative sample of young people in the UK population, sampled through a YouGov polling service panel survey, and sample weighting was incorporated into statistical analysis to obtain representative UK estimates. Nonetheless, the use of non-probability sampling is not free from biases, for example, those with existing mental health conditions may be less likely to participate in online surveys, and therefore insights from the most vulnerable may be missing.^48^ For young people aged under 17 years old, demographic information was provided at household level, that is, by parents, leading to some information (e.g. being in education, training or at work) being unavailable. The use of self-reported questionnaires may also have led to information bias, such as recall bias (e.g. COVID-19-related mortality in the family) or social desirability bias. Moreover, the cross-sectional nature of the study did not allow for an appropriate assessment of the direction and causation of significant associations. The use of validated questionnaires (e.g. PHQ-8 for depression, GAD-7 for anxiety and UCLA for loneliness) was a strength, as was the input from focus groups with young people recruited through Leaders Unlocked on questionnaire development, piloting and interpretation.

The results of the policy question at W3–5 should be interpreted with caution. There were no free text options and none of the pre-selected list of options (co-designed with young people) were endorsed by more than 40% of participants. Furthermore, an administrative error at W5 meant participants only had one option for the policy question rather than multiple ones as in previous waves.

The COVID-19 pandemic had a substantial impact on young people, whether on their mental health, their social contacts and interactions or their perspective on what the future holds for them. Young people experienced higher levels of anxiety during the summer and fall of 2020, followed by higher levels of depression during the 2020–2021 winter, with loneliness gradually increasing to peak during the spring and summer of 2021. Young people who were female, older, with pre-existing mental-health issues or experiencing financial difficulties were at higher risk of anxiety, depression and loneliness. However, help-seeking behaviours reduced the risk of depression and loneliness. Young people strongly advocated for better teacher training, and a better integration of mental health services, particularly within their schools.

## Supporting information

Rouquette et al. supplementary material Rouquette et al. supplementary material

## Data Availability

The data-sets analysed during the current study are not publicly available as per agreement in the ethical approval and participant consent to participate in the study.
