# Supplementary material for: Mental health and its wider determinants in young people in the UK during 12 months of the COVID-19 pandemic: repeated cross-sectional representative survey
Source: BJPsych Open. 2024 Dec 5;10(6):e214. doi: 10.1192/bjo.2024.726 (PMC11698137; doi:10.1192/bjo.2024.726)
Supplement: Rouquette et al. supplementary material [file S2056472424007269sup001.docx]

Supplementary Table 1 – Percentage of participants reporting that themselves or others had been affected Coronavirus infections throughout five waves of data collection from 1 (24 August 2020) to 5 (26 August 2021).

| **1**  **24 Aug 20** | **2**  **17 Nov 20** | **3**  **25 Feb 21** | **4**  **24 May 21** | **5**  **26 Aug 21** | **For the following question, we would like you to think about yourself, members of your household or other family members who have been affected by Coronavirus (COVID-19) this year....** |
| --- | --- | --- | --- | --- | --- |
| 0.7% | 3.3% | 5.7% | 5.2% | 12.1% | I have been tested for Coronavirus and had a positive result |
| 9.1% | 19.7% | 34.6% | 52.8% | 52.2% | I have been tested for Coronavirus and had a negative result |
| 2.7% | 6.9% | 11.1% | 9.6% | 16.2% | Someone in my family/household has tested positive for Coronavirus |
| 5.0% | 9.3% | 12.5% | 10.4% | 13.7% | A family member living at a different address has tested positive for Coronavirus |
| 0.5% | 1.0% | 0.6% | 0.9% | 1.0% | I am currently self-isolating with symptoms of Coronavirus |
| 1.0% | 2.1% | 1.0% | 1.0% | 1.8% | My household is currently self-isolating because one of the household members has symptoms of Coronavirus |
| 1.7% | 3.1% | 1.8% | 0.9% | 2.4% | A family member living at a different address is currently self-isolating with symptoms of Coronavirus |

Supplementary table 2 *- Results of the stepwise weighted logistic binomial regression with robust standard errors (HC3) for Anxiety, Depression, and Loneliness with odds ratios, 95% CI, and p-value, controlling for ethnicity (white versus ethnic minority group) and regions in the UK.*

|  | **Anxiety (GAD-7 >=10)** | | | **Depression (PHQ-8 >=10)** | | | **Loneliness (UCLA >=6)** | | |
| --- | --- | --- | --- | --- | --- | --- | --- | --- | --- |
| Variables | Odds Ratios | 95% CI | p | Odds Ratios | 95% CI | p | Odds Ratios | 95% CI | p |
| **Wave 1 – 24 Aug 20 (Ref.)** |  |  |  |  |  |  |  |  |  |
| Wave 2 – 17 Nov 20 | 1.02 | 0.68 – 1.55 | 0.815 | 1.22 | 0.81 – 1.84 | **0.041** | 1.03 | 0.77 – 1.39 | 0.657 |
| Wave 3 – 25 Feb 21 | 0.8 | 0.53 – 1.21 | **0.026** | 1.59 | 1.06 – 2.38 | **<0.001** | 1.08 | 0.80 – 1.46 | 0.252 |
| Wave 4 – 24 May 21 | 0.84 | 0.55 – 1.28 | 0.08 | 1.08 | 0.72 – 1.64 | 0.401 | 1.21 | 0.90 – 1.62 | **0.009** |
| Wave 5 – 26 Aug 21 | 0.87 | 0.57 – 1.33 | 0.189 | 0.98 | 0.65 – 1.48 | 0.828 | 1.21 | 0.90 – 1.62 | **0.006** |
| **13-17 years old (Ref.)** |  |  |  |  |  |  |  |  |  |
| 18-19 years old | 1.28 | 0.97 – 1.69 | **<0.001** | 2.07 | 1.58 – 2.73 | **<0.001** | 1.57 | 1.26 – 1.96 | **<0.001** |
| **Male (Ref.)** |  |  |  |  |  |  |  |  |  |
| Female | 1.39 | 1.07 – 1.82 | **<0.001** | 1.31 | 1.00 – 1.71 | **<0.001** | 1.32 | 1.09 – 1.60 | **<0.001** |
| **No mental health issue (Ref.)** |  |  |  |  |  |  |  |  |  |
| Pre-existing mental health issues | 3.23 | 2.19 – 4.79 | **<0.001** | 2.7 | 1.79 – 4.12 | **<0.001** | 1.74 | 1.16 – 2.64 | **<0.001** |
| **No financial difficulties (Ref.)** |  |  |  |  |  |  |  |  |  |
| Financial difficulties: A lot | 1.84 | 1.16 – 2.91 | **<0.001** | 2.11 | 1.32 – 3.36 | **<0.001** | 2.06 | 1.40 – 3.05 | **<0.001** |
| Financial difficulties: A little | 1.12 | 0.82 – 1.54 | 0.136 | 1.48 | 1.09 – 2.01 | **<0.001** | 1.53 | 1.23 – 1.91 | **<0.001** |
| Financial difficulties: Don't know | 1 | 0.68 – 1.48 | 0.969 | 1.32 | 0.90 – 1.92 | **0.001** | 1.37 | 1.05 – 1.79 | **<0.001** |
| Financial difficulties: Prefer not to say | 1.83 | 0.80 – 4.11 | **0.003** | 0.96 | 0.41 – 2.22 | 0.848 | 1.3 | 0.66 – 2.59 | 0.103 |
| **No social media use (Ref.)** |  |  |  | 1.05 | 0.59 – 1.91 | 0.731 | 1.12 | 0.77 – 1.64 | 0.232 |
| Social media: < 1 hour |  |  |  | 1.12 | 0.65 – 1.96 | 0.394 | 1.41 | 0.99 – 2.03 | **<0.001** |
| Social media: 1 to 4 hours |  |  |  | 1.78 | 1.00 – 3.23 | **<0.001** | 1.76 | 1.17 – 2.65 | **<0.001** |
| Social media: 4 to 6 hours |  |  |  | 2.64 | 1.44 – 4.93 | **<0.001** | 1.43 | 0.92 – 2.25 | **0.001** |
| Social media: > 6 hours |  |  |  | 0.81 | 0.32 – 2.04 | 0.365 | 1.27 | 0.66 – 2.46 | 0.121 |
| Social media: Don't know |  |  |  | 0.68 | 0.48 – 0.96 | **<0.001** | 0.72 | 0.55 – 0.95 | **<0.001** |
| **No Help-seeking behaviour (Ref.)** | 15.69 | 11.89 – 20.84 | **<0.001** |  |  |  | 4.16 | 3.12 – 5.60 | **<0.001** |
| Help-seeking Behaviour |  |  |  | 15.41 | 11.64 – 20.56 | **<0.001** | 2.29 | 1.68 – 3.15 | **<0.001** |
| Depressive disorder (PHQ-8 >=10) | 2.34 | 1.72 – 3.21 | **<0.001** | 4.25 | 3.18 – 5.73 | **<0.001** |  |  |  |
| Anxiety disorder (GAD-7 >= 10) |  |  |  |  |  |  |  |  |  |
| Loneliness (UCLA >=6) | 1.02 | 0.68 – 1.55 | 0.815 | 1.22 | 0.81 – 1.84 | **0.041** | 1.03 | 0.77 – 1.39 | 0.657 |
| Observations | 11192 | | | 11192 | | | 11192 | | |
| R^2^ Tjur | 0.435 | | | 0.48 | | | 0.217 | | |
| AIC | 64.22 | | | 72.444 | | | 142.01 | | |

Supplementary Table 3 *– Percentage and rank (multiple choice) of young people endorsing suggestions to improve their mental-health as Coronavirus restrictions ease at Wave 3 (25 Feb 2021) of data collection*

| **Percentage** | **Rank** | **Which, if any, of the following in your opinion can be done to benefit and improve your mental health as Coronavirus (COVID-19) restrictions ease?** |
| --- | --- | --- |
| 38.70% | 1 | Help teachers to better understand and address teenagers’ mental health |
| 37.30% | 2 | Make it compulsory for every school/ college to have a mental health and wellbeing policy |
| 36.50% | 3 | Focus on catching up with friends and teachers rather than catching up on missed learning when returning to school |
| 33.30% | 4 | Help parents to better understand and address teenagers’ mental health |
| 32.30% | 5 | Invest in extra resources to help young people catch up academically |
| 31.60% | 6 | Recognise and address the impact of loneliness |
| 30.80% | 7 | Provide a counsellor in every school |
| 29.60% | 8 | Invest in continuous learning (e.g. apprenticeships, university, college, diploma etc) when you leave school |
| 28.80% | 9 | Deliver more high-quality apprenticeship and training opportunities for when you leave school |
| 27.10% | 10 | Invest in more support and advice helplines for young people, including to tackle loneliness |
| 24.10% | 11 | Invest in youth centres to create more opportunities for social activities |
| 15.30% | 12 | Don't know |
| 6.30% | 13 | None of these |
| 2.40% | 14 | Prefer not to say |
| Supplementary Table 4 – *Percentage and rank (multiple choice) of young people endorsing suggestions to improve their mental-health as Coronavirus restrictions ease at Wave 4 (*24 May 2021*) of data collection.* | | |
| **Percentage** | **Rank** | **Which, if any, of the following in your opinion can be done to benefit and improve your mental health as Coronavirus (COVID-19) restrictions ease?** |
| 31.80% | 1 | A counsellor in every school and an increase in counselling services available to young people (e.g. general mental health support, bereavement support, family, friends and relationship support etc.) |
| 31.30% | 2 | Programmes to get young people into work for the first time |
| 30.50% | 3 | Changing exams to only focus on what's been learnt already |
| 25.40% | 4 | Specific support for people with learning disabilities, autism, mental health difficulties and those who have been shielding or isolated during the pandemic who are struggling or anxious returning to school and to help them get back out in society |
| 22.00% | 5 | Reassurance from the Government that jobs/university places will be available |
| 21.50% | 6 | Better access to high-quality natural environments (e.g. parks, woods, ocean, rivers, gardens, allotments, house plants) |
| 21.20% | 7 | Funding for youth clubs and community groups |
| 20.30% | 8 | Better support for young people out of education (e.g. letting them know about benefits) |
| 19.40% | 9 | The government to set out a clear vision of the future |
| 18.80% | 10 | More funding available for teachers and increased recruitment of teachers so they can take on more of a social welfare role |
| 17.00% | 11 | Action on sexual abuse and harassment |
| 15.40% | 12 | Support to help young people avoid crime |
| 15.30% | 13 | Establishing a single, trustworthy source of public health information that also tackles misinformation (e.g. around the vaccine) |
| 13.50% | 14 | Don't know |
| 11.20% | 15 | Mentoring schemes and peer support programmes |
| 9.10% | 16 | Programmes to boost social connection |
| 4.60% | 17 | None of these |
| 2.20% | 18 | Prefer not to say |
| Supplementary Table 5– *Percentage and rank (single choice) of young people endorsing suggestions to improve their mental-health as Coronavirus restrictions ease at Wave 5 (*26 Aug 21*) of data collection.* | | |
| **Percentage** | **Rank** | **Which, if any, of the following in your opinion can be done to benefit and improve your mental health as Coronavirus (COVID-19) restrictions ease?** |
| 16.20% | 1 | Don't know |
| 8.10% | 2 | Make it compulsory for every school/ college to have a mental health and wellbeing policy |
| 7.90% | 3 | Focus on catching up with friends and teachers rather than catching up on missed learning when returning to school |
| 7.90% | 3 | Invest in extra resources to help young people catch up academically |
| 7.90% | 3 | Provide a counsellor in every school |
| 7.60% | 6 | Help teachers to better understand and address teenagers' mental health |
| 7.50% | 7 | Deliver more high-quality apprenticeship and training opportunities for when you leave school |
| 6.70% | 8 | Invest in continuous learning (e.g. apprenticeships, university, college, diploma etc) when you leave school |
| 6.30% | 9 | None of these |
| 6.10% | 10 | Help parents to better understand and address teenagers' mental health |
| 6.00% | 11 | Invest in more support and advice helplines for young people, including to tackle loneliness |
| 5.60% | 12 | Invest in youth centres to create more opportunities for social activities |
| 4.00% | 13 | Recognise and address the impact of loneliness |
| 2.20% | 14 | Prefer not to say |
